# Migraine epidemiology and comorbidities in Southern Israel: a clinical database study in a universal health coverage setting

**DOI:** 10.1186/s10194-022-01513-w

**Published:** 2022-12-14

**Authors:** Ido Peles, Mohnnad Asla, Mariya Abayev, Michal Gordon, Victor Novack, Rinat Ribalov, Tamar Lengil, Ron Maor, Mayera Elizur, Gal Ifergane

**Affiliations:** 1 Clinical Research Center, Soroka University Medical Center and the Faculty of Health Sciences, Ben-Gurion University of the Negev, Beer-Sheva, Israel; 2 Department of Neurology, Brain Medicine Division, Soroka University Medical Center and the Faculty of Health Sciences, Ben-Gurion University of the Negev, Beer-Sheva, Israel; 3 Internal Medicine Division, Soroka University Medical Center and the Faculty of Health Sciences, Ben-Gurion University of the Negev, Beer-Sheva, Israel; 4 Teva Pharmaceuticals Industries Ltd, Tel Aviv, Israel

**Keywords:** Migraine, Epidemiology, Israel, Prevalence, Diagnosis

## Abstract

**Background:**

Understanding migraine epidemiology and its burden is crucial for planning health policies and interventions at the local level as well as at the global level. National policies in Israel rely on global estimations and not on local data since local epidemiologic studies had not previously been performed. In this study, we evaluated the epidemiology of migraine in the southern district of Israel using the electronic medical records database of the largest Israeli health maintenance organization (HMO).

**Methods:**

In this population-based, retrospective, observational cohort study, adult migraine patients were identified in the computerized database of the southern district of the Clalit Health Services HMO (total population, 0.75 million). Patients were identified based on recorded diagnosis (*International Classification of Diseases, Ninth Revision*) and/or claims for specific anti-migraine medication (triptans) between 2000 and 2018. A 1:2 age-, gender-, and primary care clinic–matched control group was used for evaluation of comorbidities.

**Results:**

In 2018, a total of 29,938 patients with migraine were identified out of 391,528 adult HMO members. Most of the patients were women (75.8%), and the mean ± standard deviation age at diagnosis was 36.94 ± 13.61 years. The overall prevalence of migraine (per 10,000) was 764.64 (7.65%), 1143.34 (11.43%) for women and 374.97 (3.75%) for men. The highest prevalence was observed in patients aged 50 to 60 years and 40 to 50 years (1143.98 [11.44%] and 1019.36 [10.19%], respectively), and the lowest prevalence was among patients aged 18 to 30 years and > 70 years (433.45 [4.33%] and 398.49 [3.98%], respectively).

**Conclusions:**

This is the first large-scale epidemiologic study of migraine prevalence in Israel. Compared to international estimations, migraine appears to be underdiagnosed in the southern district of Israel.

## Background

Migraine is a common disabling disease that affects millions around the globe [1]. The understanding of its epidemiology and burden is crucial for planning health policies and interventions at the local level as well as at the global level [2]. In Israel, the epidemiology of migraine was never evaluated on a large scale [3], and national policies rely on global estimations instead of local data.

The lack of biomarkers and the subjective nature of this disease have made the evaluation of its epidemiology extremely challenging, especially in the setting of limited or unequal access to health care. Many patients do not consult health care providers about their headaches, and when they do, migraine seems to be poorly diagnosed or poorly documented [4]. Decentralized health care systems and databases contribute to this challenge. Researchers have attempted to overcome this challenge by performing large-scale community surveys (using face-to-face [5], telephone [6], postal [7], or web-based [8] interviews or questionnaires) or by using data from large national statistical [9] or administrative [10] datasets. These approaches address the health care access bias of clinical databases but have inherent response bias, as patients with headache and their households probably have a higher tendency to respond to headache surveys than others. Those approaches usually rely on subjective reports, not clinician diagnosis. Moreover, such surveys are relatively expensive and require a complicated infrastructure and expertise.

Many previous studies have suggested that migraine is associated with a variety of comorbidities, particularly cardiovascular, psychiatric, neurological, and chronic inflammatory disease, but rarely have all of these comorbidities been compared between patients with and without migraine. Understanding the comorbidity of migraine is clinically important because of the bilateral risk of increased morbidity, the risk of medication overuse, and the adjustment of treatment to the comorbidities’ limitations [11].

In this study, the unique setting of an accessible and equal health care system with a centralized data system, due to the structure of the universal coverage health care system in Israel, allows us to evaluate the prevalence and comorbidities of clinically diagnosed migraine using a computerized clinical database.

## Methods

### Clinical setup

The structure of the health care system in Israel is based on a universal coverage system providing primary care through 4 health maintenance organizations (HMOs). The National Health Insurance Law mandates that all citizens residing in Israel join 1 of 4 official non-profit HMOs that are prohibited by law from denying membership. The Clalit Health Services (CHS) divides Israel into a number of geographic regions, and residents within each region have similar access to health services. To eliminate interregional heterogeneity [12], we included study patients residing in the southern district of Israel, the Negev region. The largest city in that region is Beer-Sheva, which is considered the capital of the Negev region. Overall, 8.2% of the Israeli population live in this region; 75% are Jewish and 25% are Bedouin. Municipal communities within the southern district are ethnically homogenous.

CHS, the largest Israeli HMO, is also the largest health care provider in the Negev region, covering approximately 67% of its 730,000 residents (and 50% of the total population in the country), with primary clinics available in every city, town, or settlement. Soroka University Medical Center (SUMC) is a tertiary 1100-bed medical center, with > 65,000 hospitalizations and about 200,000 emergency department visits annually, and is the largest regional hospital in southern Israel. SUMC is also a part of the HMO's hospital network. This unique setup of 1 hospital in 1 large geographic area facilitates a close follow-up and population-based assessment, with minimal referral bias, such that virtually no patients are lost to follow-up.

### Study population and data collection

We performed a population-based, retrospective, observational cohort study. Adult (≥ 18 years) patients with migraine were identified in the computerized database for the southern district of the CHS. Patients with migraine were identified based on recorded physician diagnosis (*International Classification of Diseases, Ninth Revision* [ICD-9]) of migraine (with or without aura) and/or claims for specific anti-migraine medication (triptans) between 2000 and 2018.

**Fig. 1:**
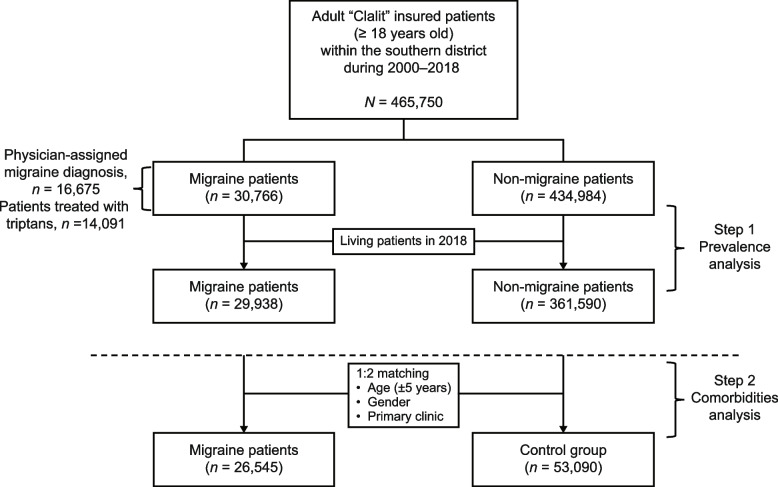
Study flow

We built our cohort in two steps: we first identified patients with a diagnosis of migraine made by a physician (16,675 patients with or without a triptan prescription), and then from those without a diagnosis made by a physician, we identified patients with a triptan prescription (14,091 patients). A high percentage of patients with a recorded diagnosis of migraine were also treated with triptans (71.8%). The study population’s flow is shown in Fig. 1.

Physician-assigned migraine diagnosis was given by either a primary care physician or neurologist. Migraine diagnoses assigned by primary care physicians were found to be highly reliable. The Landmark Study demonstrated that clinic-assigned diagnosis of migraine was validated by an expert panel based on diary data in 98% of cases [13].

Triptans are migraine-specific abortive drugs [14]. The only labeled indication for this drug class in Israel is the acute treatment of migraine. Triptans are sometimes used off-label for the treatment of cluster headache [15]; however, its prevalence (0.1% of the general population) is negligible compared to that of migraine. Therefore, we considered triptan-prescribed patients as being diagnosed with migraine even when such a diagnosis was not recorded.

Patient characteristics, including demographics (gender, age at diagnosis, ethnicity, family status, education, social state score, and immigrant status) and clinical history (comorbidities, medications, hospitalization notes, diagnostic imaging, and primary care physician visits), were collected from a central computerized database of the CHS system.

For socioeconomic characteristics, we specifically used the socioeconomic index (SEI), scoring each municipality according to 14 variables (average monthly income, vehicle class, percentage of new vehicles, percentage of people with a high school diploma, students, percentage of work searchers, percentage of people with minimal monthly income values, people with more than double the average monthly income, median age, dependency ratio, percentage of families with ≥ 4 children, percentage of unemployment benefit recipients, beneficiaries of income support, and recipients of old-age pension) on a scale of 1 to 255 (where 1 is the lowest score). Based on this score, municipalities were aggregated into SEI clusters of 1 to 10.

### Statistical analysis

We present data summaries of the main variables using descriptive statistics in the form of means and standard deviations for normally distributed quantitative variables, medians and ranges for non-normally distributed quantitative variables, and distribution in percentage for qualitative variables.

For univariate analyses, we used appropriate statistical tests. A chi-square test was used for categorical variables, with a Fisher’s exact test when needed. Continuous variables were compared using *t* tests for normally distributed variables and a Mann–Whitney U test for non-normally distributed variables. Univariate analyses were mostly used for the analysis of initial datasets that consisted of personal data records.

To inform current management practices and potential gaps in the management of migraine in this clinic population, we stratified the migraine population by the source of the migraine diagnosis (physician diagnosis and triptan prescription) and compared the rates of use of acute specific (triptan; Anatomical Therapeutic Chemical [ATC] Code NO2CC01-7) and non-specific medication (combination pain drugs including Acamol Focus, Excedrin, Migraleve, and Rokacet Plus; ATC Code NO2BE72, NO2CX50 and opioid drugs; ATC Code NO2AJ17, NO2AX02 and NO2AA55). Medication rates represent acute use at least once during the study period for each indication.

In order to estimate the diagnosis rates in southern Israel compared to the prevalence data accepted in the literature [1], we used indirect age adjustment. The study population was stratified according to age categories (5-year intervals). In each age stratum, we reported the number of observed migraine patients at the end of 2018 (end of study period), the total number of individuals (residents in the southern district insured by the CHS), the observed point prevalence of migraine per 10,000 adults, as well as the standard, age-specific prevalence of migraine per 10,000 adults derived from available tables of the reference population. The expected number of migraine patients in all age categories was calculated by multiplying the standard prevalence of migraine by the number of individuals in each age stratum. This number represents the prevalence of migraine that the study population would have experienced if it had the same age-specific prevalence rates as the reference population.

We estimated the annual migraine incidence rate per 1000 adults in the entire population over the 18-year period using the total number of incident cases in the at-risk population. Then we calculated the incidence for each interval of 5 years of age and for each gender. The population at risk of migraine comprised all subjects who had neither a recorded physician diagnosis (ICD-9) of migraine (with or without aura) nor a claim for a specific anti-migraine medication (triptans).

To compare the prevalence of comorbidity between the migraine and non-migraine samples, we used two approaches. First, we matched migraine patients 1:2 with non-migraine controls by gender, age, and primary clinic (patients are assigned to clinics based on place of residency, which correlates with socioeconomic status) and performed a univariate analysis for each comorbidity. Second, multivariable binary logistic regression models were performed to assess differences in the likelihood of each comorbid condition in the entire study population (a total of 465,750 patients) as a function of the presence of migraine diagnosis, adjusting for gender, age, and primary clinic. Odds ratios (ORs) and 95% confidence intervals (CIs) are shown along with *P* values.

For all analyses, a 2-sided *P* value < 0.05 was to be considered statistically significant. All analyses were performed using RStudio, version 1.4.1717.

### Ethics approval

The study was approved by the SUMC ethics committee, reference number 0284-19. All clinical investigations were conducted according to the principles expressed in the Declaration of Helsinki. The ethics committee approval exempted the study from informed consent due to the retrospective data collection that maintained subject confidentiality. Informed consent was waived by the institutional review board at SUMC. Patient records were anonymized and deidentified prior to analysis.

## Results

### Demographic characteristics

During the study period, a total of 465,750 Clalit-insured patients (≥ 18 years old) within the southern district were included in the analysis. Most of the population was Jewish (77.8%), and the rest was Bedouin. Immigrants (not native to Israel) comprised 40.5% of the population.

From the total population, we identified a total of 30,766 migraine patients. Migraine patients were more likely than non-migraine controls to be women (75.5% vs 48.5%, respectively; *P* < 0.001), Jewish (79.9% vs 77.7%; *P* < 0.001), Israeli born (68.6% vs 58.9%; *P* < 0.001), married (75.9% vs 67.4%; *P* < 0.001), and educated (> 12 education years; 35.8% vs 26.3%; *P* < 0.001). Table 1 presents the features of the study population.

**Table 1:** Negev Population Demographic Characteristics

Variable		Migraine patients (*n* = 30,766)	Non-migraine controls (*n* = 434,984)	Overall (*N* = 465,750)	P^*^
Female, *n* (%)		23,230 (75.5%)	211,077 (48.5%)	234,307 (50.3%)	< 0.001
Ethnicity, *n* (%)	Jewish	24,577 (79.9%)	337,847 (77.7%)	362,424 (77.8%)	< 0.001
	Bedouin	6028 (19.6%)	96,372 (22.2%)	102,400 (21.9%)	
Age at diagnosis, median (IQR) mean (SD)	Overall	34.46 (25.45–46.49) 37.65 (14.32)	—	—	
	Female	35.03 (25.61–46.73) 37.74 (14.06)	—	—	
	Male	32.78 (25.09–45.68) 37.38 (15.10)	—	—	
Family status, n (%)	Married	23,361 (75.9%)	293,436 (67.4%)	317,836 (68.2%)	< 0.001
	Single	3473 (11.2%)	59,686 (13.7%)	62,754 (13.4%)	
	Divorced	2563 (8.3%)	30,449 (7.0%)	33,235 (7.1%)	
	Widowed	1612 (5.2%)	51,411 (11.8%)	51,923 (11.1%)	
Education, n (%)	Elementary	4856 (15.7%)	106,522 (24.4%)	110,095 (23.6%)	< 0.001
	High school	14,889 (48.3%)	213,672 (49.1%)	228,454 (49.0%)	
	Tertiary	11,020 (35.8%)	114,789 (26.3%)	127,200 (27.3%)	
SEI score, mean (SD)		8.87 (3.94)	8.36 (3.86)	8.40 (3.86)	< 0.001
Immigrant, *n* (%)		9674 (31.4%)	178,846 (41.1%)	188,520 (40.5%)	< 0.001

*IQR* Interquartile range, *SD* Standard deviation, *SEI* Socioeconomic index

^*^Chi-square test for categorical variables/*t* test for normally distributed variables

**Fig. 2:**
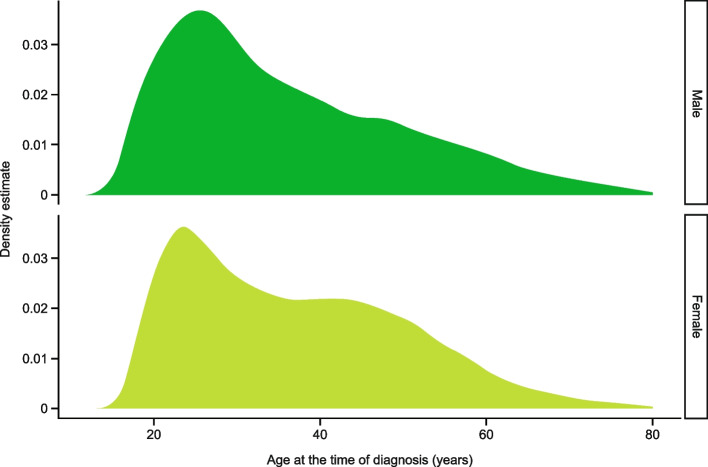
Migraine diagnosis age by gender

The median age of onset was slightly higher among female patients than male patients (median [interquartile range], 35.03 [25.61–46.73] vs 32.78 [25.09–45.68]; *P* = 0.03). This is explained by the fact that there is a prevalence peak around the age of 25 years in both groups, but there is bimodal distribution with another minor prevalence peak around the age of 45 years in the female group only (Fig. 2).

Table 2 presents acute specific and non-specific medication use rates, based on medication use at least once during the study period, for each indication. We stratified the migraine population by the source of the migraine diagnosis (physician diagnosis and triptan prescription). There is a high percentage of patients with a recorded diagnosis of migraine treated with triptans (71.8%).

**Table 2:** Migraine Population - Drug Consumption

Variable, *n* (%)	Physician-assigned diagnosis patients (*n* = 16,675)	Triptan-prescribed patients (*n* = 14,091)	Overall (*n* = 30,766)
Triptan	11,973 (71.8%)	14,091 (100.0%)	26,064 (84.7%)
Combination pain drug	6721 (40.3%)	5890 (41.8%)	12,611 (41.0%)
Opioid drug	5302 (31.8%)	4424 (31.4%)	9726 (31.6%)

### Prevalence analysis

**Fig. 3:**
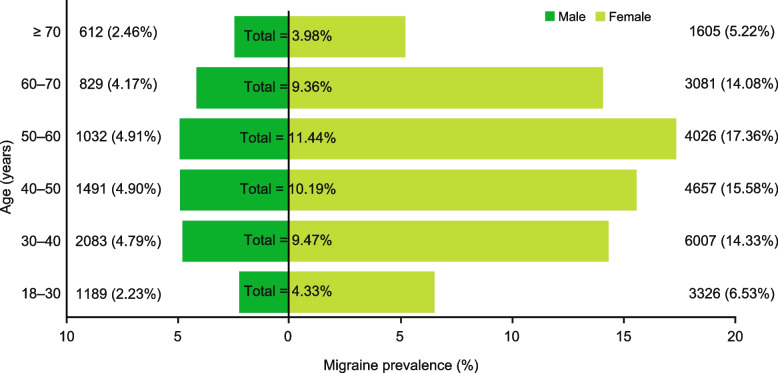
Standard age pyramid for migraine prevalence in 2018

The point prevalence of migraine was calculated. A total of 29,938 patients with migraine who were alive and still insured by the CHS at the end of 2018 were identified out of 391,528 adult HMO members. The overall migraine diagnosis prevalence rate was 7.65% for the total population, 11.43% for women, and 3.75% for men. The highest migraine diagnosis prevalence was observed in the age groups of 50 to 60 years and 40 to 50 years (11.44% and 10.19%, respectively), and the lowest diagnosis prevalence was observed among the populations aged 18 to 30 years and > 70 years (4.33% and 3.98%, respectively; Fig. 3).

As shown in Table 3, after we calculated the prevalence for 2018 per 10,000 adults and then for each interval of 5 years of age, we adjusted the prevalence data accepted in the literature that show an age-standardized prevalence of 14.4% worldwide [1] for the age distribution of our study population, which allowed us to estimate the diagnosis rates in southern Israel. The estimated prevalence expected for our study population in the southern district of Israel was 17.38% after indirect age adjustment according to the prevalence data accepted in the literature.

**Table 3:** Age-standardized Prevalence in 2018

Age, years	Observed migraine patients	Expected migraine patients	Observed prevalence, %	Expected prevalence, %
18–19	128	2067.06	1.09%	17.58%
20–24	1438	9063.77	3.05%	19.24%
25–29	2949	9190.36	6.51%	20.29%
30–34	3950	9253.19	9.02%	21.14%
35–39	4140	8923.05	9.93%	21.41%
40–44	3369	7564.33	9.60%	21.56%
45–49	2779	5063.06	11.02%	20.07%
50–54	2555	3966.67	11.74%	18.23%
55–59	2503	3680.37	11.15%	16.39%
60–64	2272	3124.92	10.24%	14.08%
65–69	1638	2296.70	8.37%	11.74%
70–74	1072	1440.39	7.05%	9.47%
75–79	582	830.59	5.56%	7.93%
80–84	333	626.92	3.66%	6.89%
85–89	163	369.97	2.68%	6.08%
90–94	44	187.80	1.17%	5.00%
≥ 95	23	407.41	0.21%	3.70%
Total	29,938	68,056.53	7.65%	17.38%

**Fig. 4:**
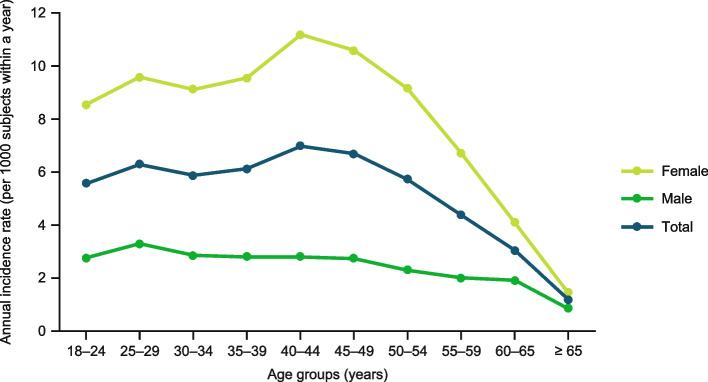
Annual incidence (per 1000 subjects within a year) rates in age groups

Figure 4 shows the estimated annual age-specific incidence rates for both women and men. The overall migraine incidence was estimated at 4.97 per 1000 subjects within a year (18-year longitudinal study); the incidence was 7.49 per 1000 women within a year and 2.44 per 1000 men within a year. Migraine incidence peaked between the ages of 40 to 44 years in women (11.18 per 1000 subjects within a year) and the ages of 25 to 29 years in men (3.29 per 1000 subjects within a year). Finally, the fluctuations around the average annual incidence based on the 18-year study period are small (4.27–5.71).

### Comorbidities analysis

For the analysis of comorbidities, we compared migraine patients to non-migraine controls matched by gender, age, and primary clinic (patients are assigned to clinics based on place of residency, which correlates with socioeconomic status). After matching patients with migraine to non-migraine controls, we had 26,545 patients with migraine and 53,090 non-migraine controls we were able to match.

Several conditions were found to be more prevalent among patients with migraine than among non-migraine controls. These conditions included pain disorders, such as low back pain (migraine patients, 10.3% vs non-migraine controls, 9.0%; *P* < 0.001) and fibromyalgia (6.6% vs 4.0%, respectively; *P* < 0.001); psychiatric disorders, such as anxiety (3.6% vs 1.9%; *P* < 0.001) and depression (3.2% vs 1.1%; *P* < 0.001); and cardiovascular risk factors, such as hypertension (20.2% vs 11.9%; *P* < 0.001) and dyslipidemia (28.1% vs 25.5%; *P* < 0.001; Table 4).

**Table 4:** Comorbidities

Variable, *n* (%)	Migraine patients (*n* = 26,545)	Non-migraine controls^*^ (*n* = 53,090)	*P*^†^
**Psychiatric disorders**			
Anxiety	956 (3.6%)	1009 (1.9%)	< 0.001
Depression	849 (3.2%)	584 (1.1%)	< 0.001
**Cardiovascular risk factors**			
Obesity (BMI > 30 kg/m^2^)	8335 (31.4%)	20,280 (38.2%)	< 0.001
Dyslipidemia	7459 (28.1%)	13,538 (25.5%)	< 0.001
Essential hypertension	5362 (20.2%)	6318 (11.9%)	< 0.001
Diabetes mellitus	2708 (10.2%)	7433 (14.0%)	< 0.001
Pre-diabetes	1141 (4.3%)	3026 (5.7%)	< 0.001
**Pain disorders**			
Fibromyalgia	1752 (6.6%)	2124 (4.0%)	< 0.001
Low back pain	2734 (10.3%)	4778 (9.0%)	< 0.001
Disc lesions	1274 (4.8%)	2071 (3.9%)	< 0.001
**Other**			
Hypothyroidism	2071 (7.8%)	4300 (8.1%)	0.249
Vitamin D deficiency	1858 (7.0%)	3769 (7.1%)	0.616
Asthma	1832 (6.9%)	3238 (6.1%)	0.238
Fatty liver	1566 (5.9%)	2973 (5.6%)	0.223
Vitamin B12 deficiency	1646 (6.2%)	3292 (6.2%)	0.912
Osteoporosis	1566 (5.9%)	3026 (5.7%)	0.430
Iron deficiency anemia	903 (3.4%)	2283 (4.3%)	< 0.001
Annual mortality (2018)	70 (0.3%)	186 (0.4%)	0.717

*BMI* body mass index

^*^The non-migraine control group is matched to the migraine group based on gender, mean age (± 5 years), and primary clinic

^**†**^Chi-square test for categorical variables/*t* test for normally distributed variables

By contrast, obesity (body mass index > 30 kg/m^2^; 31.4% vs 38.2%, respectively; *P* < 0.001), diabetes mellitus (10.2% vs 14.0%; *P* < 0.001), and pre-diabetes (4.3% vs 5.7%; *P* < 0.001) were less common among patients with migraine compared with non-migraine controls (Table 4).

**Fig. 5:**
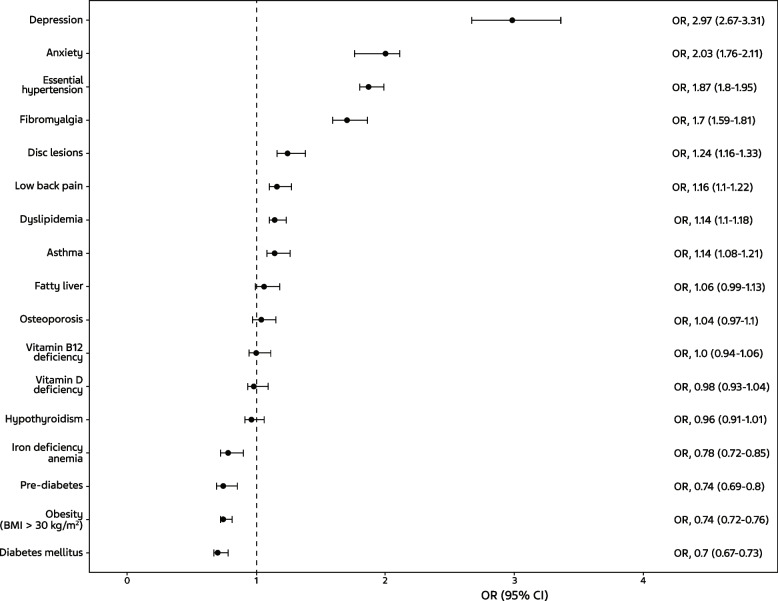
Relative odds of migraine (and 95% CI) versus non-migraine controls for each comorbid condition. CI, confidence interval; OR, odds ratio

Figure 5 provides the OR and 95% CI for the migraine cohort versus the non-migraine cohort in the entire study population (a total of 465,750 patients) for each health condition, adjusting for gender, age, and primary clinic. For all psychiatric and pain disorders, the ORs were in the same direction, showing greater risk for the migraine cohort. After adjusting, patients with migraine compared to the non-migraine cohort were almost three times more likely to experience depression (OR [95% CI], 2.97 [2.7–3.3]). The migraine group was at least twice as likely to experience anxiety (OR [95% CI], 2.03 [1.8–2.1]) and more than one and a half times more likely to experience hypertension (OR [95% CI], 1.87 [1.8–2.0]) and fibromyalgia (OR [95% CI], 1.70 [1.6–1.8]). For several metabolic conditions, including obesity, diabetes mellitus, and pre-diabetes, the ORs were in the opposite direction, showing greater risk for the non-migraine cohort.

## Discussion

In this study, 29,938 adult patients who were ever assigned a diagnosis of migraine and/or treated with triptans were identified, comprising 7.65% of the patients covered by the southern district of the CHS HMO in 2018. The prevalence of migraine was found to be higher in women (11.43%) compared to men (3.75%) in a ratio of 3:1. The median age of migraine diagnosis in our cohort was 34.5 years.

Although our study uses clinical data rather than community survey results and cannot be compared to other epidemiologic studies, several comparisons can be made. This 1-year prevalence is much lower than the global estimation [1, 16] of 14% (ranging from 9%–35% in different countries), while the gender ratio is similar to that previously reported.

Migraine epidemiology studies typically focus on prevalence and, to our knowledge, its incidence has been directly evaluated in only two studies [17, 18]. We found an incidence of 4.97 per 1000 subjects within a year (18-year longitudinal study), which was significantly lower than previously reported (8.1–23.8 per 1000 subjects within a year).

Migraine has repeatedly been found to be underdiagnosed and misdiagnosed [19]. The relatively low prevalence of migraine (based on clinical database methods that rely on diagnosis and medication prescriptions) in our cohort possibly represents this discrepancy. Patients who never consulted a physician regarding their headaches and patients who were misdiagnosed are not included in our study. This probably explains the big discrepancy between our findings and those of previously reported epidemiologic studies and limits the generalizability of our study findings only to the consulting and treated population of patients with migraine.

The different methodology used in our study is also reflected in the older age of peak incidence, which was 40 to 44 years in our study compared to 25 to 34 years in previous reports. One study found that the onset of migraine was before age 25 years [20] in 50% of migraine patients, while the median age of actual diagnosis in our study was found to be approximately 35 years. It is reasonable to speculate that this discrepancy represents delays of approximately a decade in the diagnosis of migraine in patients in the southern district of Israel.

The design of this study allowed us to examine migraine comorbidities in a large, valid clinical database. Three categories of comorbidities were found: psychiatric disorders, pain disorders, and cardiovascular risk factors.

The association between migraine and both depression and anxiety disorders has been established in several studies [21–23]. Our study found a similar association, with higher rates of depression (3.2% vs 1.1%, respectively) and anxiety (3.6% vs 1.9%) among migraine patients compared to matched controls.

We found that both fibromyalgia and low back pain were associated with migraine (6.6% vs 4.0% and 10.3% vs 9.0%, respectively); several studies have reported such comorbidities previously [23–25].

While hypertension and dyslipidemia were found to be more prevalent among patients with migraine than matched controls (20.2% vs 11.9% and 28.1% vs 25.5%, respectively), the prevalence of diabetes, pre-diabetes, and obesity was found to be lower (10.2% vs 14.0%, 4.3% vs 5.7%, and 31.4% vs 38.2%, respectively). The association of diabetes and migraine was evaluated in several studies and yielded inconclusive results [23, 26]. The finding of lower diabetes rates in migraine patients in our cohort has not been previously reported.

Our study is the most comprehensive to report on migraine epidemiology in Israel. Although it does not reflect the true prevalence of the disease, it provides valuable information for clinicians and national policy makers, presenting the burden of migraine in the health care system and providing a basis for decisions on health resource allocation. The use of available databases allows easy replication in other regions and HMOs and adds a clinical validity to the case definition of migraine and its comorbidities.

The weakness of this study design is its underestimation of migraine prevalence. It is possible that not all patients included in this study population were correctly diagnosed. Given that the current results support the underdiagnosis of migraine in the study population, increasing the specificity of diagnosis would likely only strengthen that finding. In addition, the medical records did not capture the severity of migraine (since headache days are not registered in the clinical database) or the distinction between episodic and chronic migraine. Although migraine diagnosis and triptan prescriptions were assigned by non-specialists, it is reasonable to assume underdiagnosis and not overdiagnosis. However, the structure of the health care delivery system that provides universal access to primary clinics, consultants, and hospital care, combined with the complete availability of medical records at all levels of care, gives us a unique opportunity to study the epidemiology of clinically validated migraine in a large population.

Comparing community surveys with clinical databases could allow us to understand the gaps between the burden of migraine and migraine care and should be investigated in future studies. Health care systems should focus on complete and correct diagnosis of migraine through public health awareness campaigns, primary care, and specialist services to ensure that all migraine patients receive adequate care and reduce the burden to society.

## Data Availability

The datasets analyzed during the current study are available from the corresponding author on reasonable request.

## Abbreviations

CHS: Clalit Health Services
CI: Confidence interval
HMO: Health maintenance organization
ICD-9: *International Classification of Diseases, Ninth Revision*
IQR: Interquartile range
OR: Odds ratio
SD: Standard deviation
SEI: Socioeconomic index
SUMC: Soroka University Medical Center
